# Application of intervention of information motivation behavior skill (IMB) model in the cardiac rehabilitation of patients with coronary heart disease

**DOI:** 10.12669/pjms.38.6.5721

**Published:** 2022

**Authors:** Meihua Li, Hailing Zhu

**Affiliations:** 1 Meihua Li, Department of Cardiology, The First People’s Hospital of Chenzhou, Chenzhou 423000, Hunan Province, P.R. China; 2 Hailing Zhu, Department of Cardiology, The First People’s Hospital of Chenzhou, Chenzhou 423000, Hunan Province, P.R. China

**Keywords:** Behavior change integration theory, Motivational intervention, Coronary heart disease, Cardiac rehabilitation

## Abstract

**Objectives:**

To explore the effects of information motivation behavior skill (IMB) model in the cardiac rehabilitation of patients with coronary heart disease.

**Methods:**

We analyzed data from medical records of patients with coronary heart disease who had received cardiac rehabilitation treatment in the Department of Cardiology of our hospital from April 2019 to May 2021. Data from 52 patients who had received routine nursing rehabilitation (Mode-I) and 56 patients that received IMB model-oriented nursing rehabilitation (Mode-II) was included. We calculated indexes of cardiopulmonary function, physical fitness, and nursing satisfaction, and self-efficacy scores (GSEs) before and three months after the intervention for patients in the two rehabilitation groups.

**Results:**

We found similar LVEFs, SVs, anaerobic thresholds, peak oxygen uptake scores, PEFs, and FVCs in patients undergoing either of the two rehabilitation modes before the interventions (P>0.05). However, these indexes were significantly higher in patients undergoing Mode-II rehabilitation after the intervention, compared to Mode-I group (P<0.05). We found similar aerobic endurances, upper limb and lower limb muscle strengths in patients undergoing either method before the intervention (P>0.05). After the treatment, these parameters were higher in the Mode-II group compared to Mode-I group (P<0.05). The scores of daily life behavior management, cognitive symptom management, and disease management between the two groups were similar before the intervention (P>0.05). After the intervention, patients undergoing Mode-II rehabilitation had significantly higher values than patients in the Mode-I group (P<0.05). The nursing satisfaction of the patients in the Mode-II group (94.64%) was significantly higher than that of patients in the Mode-I group (80.77%) (P<0.05).

**Conclusion:**

IMB model intervention measures can improve cardiopulmonary function and physical fitness, and enhance the patients’ self-efficacy, resulting in high nursing satisfaction.

## INTRODUCTION

Prevalence of coronary heart disease is on the rise with the increased aging of the general population and the adoption of unhealthy habits.^1^,^2^ While the mortality, associated with the coronary heart disease, has decreased significantly due to the advances in the medical care, even after the successful treatment, some patients are at increased risk of coronary restenosis. Management of these patients, therefore, is not limited to symptom relief and reducing mortality, but rather requires attention to overall health variables.^3^,^4^

Cardiac rehabilitation promotes social, psychological, and physiological well-being of patients with heart disease through different intervention measures designed to reduce mortality and improve the quality of life.^5^ Effective nursing interventions during cardiac rehabilitation for patients with coronary heart disease helps patients to better understand their own condition and promotes their active cooperation during the rehabilitation treatment.^6^ Information motivation behavior skills (IMB) model is an important clinical intervention model. It posits that patients must be informed, motivated, and behaviorally skilled to adopt, implement and maintain positive health behaviors.^7^,^8^ Using motivational interviews, IMB model helps to address and clarify current problems of the patients, and provides them with the available support resources and information to change their own bad health habits.^9^ Based on this, we extracted clinical data from records of 108 patients with coronary heart disease who had undergone cardiac rehabilitation at our hospital, and we retrospectively analyzed and summarized the nursing schemes and relevant rehabilitation indicators to evaluate the rehabilitation interventions.

## METHODS

We analyzed data from 108 patients (59 men and 49 women) with coronary heart disease who underwent cardiac rehabilitation treatment in the Department of Cardiology of our hospital from April 2019 to May 2021.

### Inclusion criteria:

• The patients met the diagnostic criteria for coronary heart disease.^10^
• Have basic reading, writing, and comprehension skills.
• Vision and hearing will not affect oral and written communication.
• Mobility.
• Age over 18 years.
• Stable condition.
• NYHA classification of cardiac function - grade II-III.

### Exclusion criteria:

• Patients with severe arrhythmia.
• Patients with end-stage disease.
• Patients unable to tend to themselves.
• Patients with psychosis.
• Patients with benign or malignant tumors.
• Patients with organic lesions to the kidney, liver, or other organs.

### Ethics Approval

The medical ethics committee of our hospital approved this study (Approval number: CZDYRMYY21122, Date: 2021-June-27), and all patients were informed and agreed to participate freely.

Patients were retrospectively divided into two groups (Mode-I and Mode-II) based on the nursing strategy. For a routine nursing scheme (Mode-I), patients were provided with health knowledge manuals. Medical professionals held education sessions for patients, including basic knowledge and treatment measures for coronary heart disease and cardiac rehabilitation measures; the material also included the positive significance of changing unhealthy lifestyles, guided patients on how to effectively monitor heart rate and blood pressure, and encouraged them to actively participate in their own cardiac rehabilitation

The rehabilitation intervention was initiated when the patient’s vital signs were stable (normal blood pressure and body temperature, resting heart rate <100 beats/min), exercises were carried according to each patient’s status and tolerance (up- and downstairs activities, sitting defecation, transition from sitting to walking exercises, breathing exercises and distal limb exercises in bed) to encourage the patient to quit smoking and alcohol.

Patients were given routine discharge guidance, and they were told to continue functional rehabilitation exercises outside of the hospital setting. After discharge, patients were regularly followed up by telephone to help them manage their out-of-hospital cardiac rehabilitation, life, and rest status, and to guide them with any problems they encountered.

IBM model (Mode-II) was carried out on the basis of Mode-I interventions that included the following:

1) Information: face-to-face interviews with patients to assess their adherence to cardiac rehabilitation exercises, diet, medication, evaluate their psychology and risk factor management.

2) Motivation: motivational interviews, including: (1) First interview to find the source of motivation. The first interview content was determined by medical staff after consulting relevant data and integrating previous clinical experience. The interview content was finalized after the review and evaluation by the intervention team members, and the interview theme was determined as “analyzing the patients’ cardiac rehabilitation behavior, summarizing and analyzing the motivation sources that may lead to behavior change and persistence”. The specific contents of the interview were: “on a scale of 1 to 10 (the higher the score, the more important it is), how important do you think cardiac rehabilitation is to you”, “what are your wishes or goals in your daily life or work”, “have you discussed your feelings of rehabilitation with other patients with coronary heart disease in the past” “What kind of help did the community hospital or community neighborhood committee or family members provide after you suffered from coronary heart disease? What do you think of the impact of such help on you”, “what impact do you think effective cardiac rehabilitation can bring”, “what do you think of the prognosis of coronary heart disease”; (2) The second interview was conducted two days after the first interview. According to the “importance score” of patients’ cardiac rehabilitation behavior motivation, different conversation topics were selected, and different conversation strategies were adopted; (3) About five days after the second interview, the third interview was conducted to ask about the behavior improvement of patients with coronary heart disease, and select the corresponding conversation strategy in combination with the score of “importance score”, as shown in Fig.1.

3) Behavioral skills, focusing on sports rehabilitation guidance. Patients with good exercise habits got assistance in adjusting activity frequency, time and intensity, and how to exercise safely. In patients without exercise habits, their willingness and ability to exercise was assessed, and they were guided them to exercise regularly (stability exercise, flexibility stretching, strength training, heart exercises, fitness equipment, outdoor walking, etc). The first intervention was conducted before the discharge from the hospital. The intervention duration was 30 ~ 40 minutes. Another intervention was conducted at two weeks, one month, two months and three months after the discharge to further increase the effectiveness of the intervention in combination with the previous interview and the patient’s second and third medication evaluation. Each intervention lasted 20 ~ 40 minutes.

### Observation Indices

Three months after the intervention, a quark PFT exercise cardiopulmonary function test system (Cosmod company, Italy) was used to compare cardiopulmonary functions (left ventricular ejection fraction [LVEF], stroke output [SV], anaerobic threshold, peak oxygen uptake, maximum expiratory flow [PEF], and forced vital capacity [FVC]) of the two groups of patients before and after the intervention. Functional physical fitness variables (aerobic endurance, upper limb muscle strength [30s bending arm test], and lower limb muscle strength [30s sitting and standing test]) of the patients in the two groups before and after the intervention were measured. Self-efficacy scores of the patients in the two groups before and after the intervention were calculated according to the GSES scale. These scores evaluated three dimensions: daily life behavior management, cognitive symptom management, and disease management (the higher the GSES score, the better). The nursing satisfaction of the two groups was assessed based on the Newcastle Satisfaction with Nursing Scale (NSNS) with a total of 95 points. Scores lower than 67 points indicated dissatisfaction, scores between 67 and 85 indicated moderate satisfaction, and scores higher than 85 indicated high satisfaction (moderate satisfaction + high satisfaction)/total number of cases×100%=nursing satisfaction).

### Statistical Analysis

We analyzed the data using Spss22.0, and expressed measurement data as means and standard deviations (*x̅*±s), independent sample t-test was performed for inter group comparison and paired t-test was performed for intra group comparison. We expressed counting data as counts and percentages [n (%)] and processed them by *χ^2^* inspection. We considered P<0.05 as indicative of statistically significant differences.

## RESULTS

We analyzed data from 108 patients who met the inclusion criteria. Of them, 52 patients received routine nursing rehabilitation (Mode-I) and 56 received motivational intervention guided by behavior change integration theory (Mode-II). We found similar basic clinical characteristics between the two groups (P>0.05; Table-I). There was no difference in the LVEF, SV, anaerobic threshol, peak oxygen uptakes, PEF, and FVC between the two rehabilitation groups before the intervention (P>0.05). After the intervention, these indexes improved in both groups as compared to before the intervention. Patients in the Mode-II showed significantly higher LVEF, SV, anaerobic threshold, peak oxygen uptake, PEF, and FVC than Mode-I (P<0.05; Table-II).

**Table-I:** Comparison of basic characteristics of patients undergoing different rehabilitation methods.

Mode	n	Sex (M/F)	Age (years)	NYHA classification of cardiac function		Education level		

				II	III	Junior high school and below	High school	College degree or above
Mode-I	52	31/21	54.79±13.27	34 (65.38)	18 (34.62)	18 (34.62)	21 (40.38)	13 (25.00)
Mode-II	56	34/22	56.20±13.80	31 (55.36)	25 (44.64)	22 (39.28)	24 (42.86)	10 (17.86)
t		0.014	0.540	1.131		0.844		
P		0.907	0.590	0.287		0.656		

**Table-II:** Comparison of cardiopulmonary function in patients un two rehabilitation methods (*x̅* ±s).

Status	Mode	n	LVEF (%)	SV (ml)	Anaerobic threshold [ml/ (kg/min)]	Peak oxygen uptake [ml/ (kg/min)]	PEF(L/s)	FVC(L)
Before intervention	Mode-I	52	53.73±4.86	63.17±4.18	9.50±2.07	12.31±2.91	1.87±0.26	1.36±0.20
	Mode-II	56	53.27±5.22	62.62±4.69	9.26±2.55	12.10±2.74	1.91±0.22	1.39±0.21
	t		0.476	0.639	0.520	0.369	0.871	0.719
	P		0.635	0.524	0.604	0.713	0.386	0.414
After intervention	Mode-I	52	58.94±4.29	69.71±3.61	11.92±1.70	19.73±3.53	2.36±0.34	2.26±0.29
	Mode-II	56	60.80±4.41	72.86±4.16	13.55±2.21	23.64±3.65	2.99±0.42	2.87±0.32
	t		2.221	4.181	4.317	5.899	8.587	10.116
	P		0.028	<0.001	<0.001	<0.001	<0.001	<0.001

We found similar aerobic endurance, upper limb muscle strength, and lower limb muscle strength in the two groups before the intervention (P>0.05). After the intervention, both groups showed improvement compared to the values before the treatment. Mode-II rehabilitation was associated with significantly higher improvement of aerobic endurance, and upper and lower limb muscle strength compared to Mode-I (P<0.05; Table-III). Before the intervention, the scores of daily life behavior management, cognitive symptom management, and disease management were similar between the two groups of patients (P>0.05), and showed marked improvement compared to the scores before the intervention. Scores of Mode-II patients were significantly higher than those of Mode-I (P<0.05; Table-IV). The nursing satisfaction of patients undergoing Mode-II rehabilitation (94.64%) was significantly higher than that of patients undergoing Mode-I rehabilitation (80.77%) (P<0.05; Table-V).

**Table-III:** Comparison of functional physical fitness in patients undergoing different rehabilitation prescriptions (*x̅*±s).

Status	Mode	n	Aerobic endurance (m/6min)	Upper limb muscle strength (PCS/30s)	Lower limb muscle strength (PCS/30s)
Before intervention	Mode-I	52	420.44±41.22	11.56±2.90	9.63±2.42
	Mode-II	56	422.84±43.09	11.32±2.95	9.53±2.58
	t		0.295	0.419	0.205
	P		0.769	0.676	0.838
After intervention	Mode-I	52	491.44±41.15	14.11±3.94	12.23±2.88
	Mode-II	56	562.80±45.00	16.69±3.60	15.19±3.29
	t		8.488	3.773	4.964
	P		<0.001	<0.001	<0.001

**Table-IV:** Comparison of GSEs scores in patients undergoingdifferent rehabilitation methods (*x̅*±s, score).

Status	Mode	n	Daily life behavior management	Cognitive symptom management	Disease management
Before intervention	Mode-I	52	11.13±3.39	10.19±3.19	17.67±4.56
	Mode-II	56	11.23±3.72	9.80±3.13	17.58±5.30
	t		0.142	0.638	0.088
	P		0.888	0.525	0.93
After intervention	Mode-I	52	16.17±4.61	16.94±3.94	25.48±4.86
	Mode-II	56	18.73±4.66	19.75±3.76	29.71±6.32
	t		2.865	3.787	3.915
	P		0.005	<0.001	<0.001

**Table-V:** Comparison of nursing satisfaction in patients undergoing different rehabilitation methods [n (%)].

Mode	n	Very satisfied	General satisfaction	Dissatisfied	Nursing satisfaction
Mode-I	52	22 (42.71)	20 (38.46)	10 (19.23)	42 (80.77)
Mode-II	56	34 (60.71)	19 (33.93)	3 (5.36)	53 (94.64)
Χ^2^					4.901
P					0.027

## DISCUSSION

Our study demonstrated that intervention of information motivation behavior skill (IMB) model of rehabilitation was associated with improved indexes of cardiopulmonary function, functional physical fitness and GSE scores, as compared to patients who received a routine Mode-I rehabilitation (P<0.05). Therefore, the intervention of IMB model seems to have a high application value for the cardiac rehabilitation of patients with coronary heart disease, and can efficiently improve patients’ self-efficacy, cardiopulmonary function, and functional physical fitness. The research of Zarani F et al.^11^ also reported that IMB model led to higher compliance in coronary artery bypass grafting. Sheeran P et al.^12^ analyzed the results of 204 experimental tests and found that the changes in attitude and self-efficacy led to moderate changes in intention and small to moderate changes in behavior. Thus, intervention measures that are aimed to change attitudes, norms, and self-efficacy promote health behavior changes. Bluemann SM et al.^13^ analyzed 14 randomized controlled trials and showed that the extensive use of behavior theory can improve the intervention effects. In addition, Beckie TM et al.^14^ conducted a study on the physical and psychosocial changes in women with coronary heart disease after theory-driven behavior intervention and showed that the cross-theoretical model of behavior changes and the principle of motivational interview guided the development and implementation of stage-matched individualized interventions and promoted healthy changes (with resulting physical and mental health improvements). Our results are in agreement with these observations. IMB model intervention addresses pathophysiology, psychological needs, current problems and existing problems of patients, formulates intervention plans according to the evaluation results, provides disease-specific information and behavior-specific information, explores social or personal motivation resources, strengthens internal motivation, and promotes patients to consciously integrate their own motivation resources, rehabilitation behavior and psychological expectations. It aims to encourage patients to start the program and maintain the enthusiasm and willingness to continue and implement certain behavior. IMB model intervention also provides targeted medication, emotional support, exercise, diet and risk-factor-control skills and behaviors.^14^,^15^ Through the IMB model, the psychological state of patients can be improved through effective motivational interview, which provides a good basis for a follow-up intervention. The motivational interview focuses on the patient and adopts the form of guided consultation. It can stimulate the motivation of individual behavior transformation through participation and sharing, while respecting patients’ autonomy, to further improve the effect of rehabilitation.^16^,^17^

The results of this study showed that the nursing satisfaction of patients undergoing Mode-II intervention was higher than that of the patients undergoing the Mode-I intervention. Shen W et al.^19^ showed that information motivation behavioral skills model (IMB) significantly improved nursing satisfaction, medication compliance, self-care ability and quality of life of patients with aplastic anemia, which is consistent with the results of our study.

### Limitation of the study

We did not study the impact of the intervention of IMB model on the long-term rehabilitation outcomes of patients with coronary heart disease. Moreover, this is a single center study with small sample size and other studies need to confirm our findings.

## CONCLUSION

Intervention of information motivation behavior skill (IMB) model in patients with coronary heart disease undergoing cardiac rehabilitation are beneficial for improving cardiopulmonary function and functional physical fitness. It is associated with self-efficacy enhancement, and high nursing satisfaction scores. Our results may serve as a reference for healthcare professionals.

### Authors’ contributions:

**ML:** Conceived and designed the study.

**ML & HZ:** Collected the data and performed the analysis.

**ML:** Was involved in the writing of the manuscript and is responsible for the integrity of the study.

All authors have read and approved the final manuscript.
